# Computational method of the cardiovascular diseases classification based on a generalized nonlinear canonical decomposition of random sequences

**DOI:** 10.1038/s41598-022-27318-0

**Published:** 2023-01-02

**Authors:** Igor Atamanyuk, Yuriy Kondratenko, Valerii Havrysh, Yuriy Volosyuk

**Affiliations:** 1 Warsaw University of Life Science, Nowoursynowska Str. 166, 02-787 Warsaw, Poland; 2 Mykolayiv National Agrarian University, Georgi Gongadze Str. 9, Mykolaiv, 54020 Ukraine; 3 Petro Mohyla Black Sea National University, 68th Desantnykiv Str. 10, Mykolaiv, 54003 Ukraine

**Keywords:** Mathematics and computing, Applied mathematics, Computational science, Computer science, Statistics

## Abstract

Decision support systems can seriously help medical doctors in the diagnosis of different diseases, especially in complicated cases. This article is devoted to recognizing and diagnosing heart disease based on automatic computer processing of the electrocardiograms (ECG) of patients. In the general case, the change of the ECG parameters can be presented as a random sequence of the signals under processing. Developing new computational methods for such signal processing is an important research problem in creating efficient medical decision support systems. Authors consider the possibility of increasing the diagnostic accuracy of cardiovascular diseases by implementing of the new proposed computational method of information processing. This method is based on the generalized nonlinear canonical decomposition of a random sequence of the change of cardiogram parameters. The use of a nonlinear canonical model makes it possible to significantly simplify the maximum likelihood criterion for classifying diseases. This simplification is provided by the transition from a multi-dimensional distribution density of cardiogram parameters to a product of one-dimensional distribution densities of independent random coefficients of a nonlinear canonical decomposition. The absence of any restrictions on the class of random sequences under study makes it possible to achieve maximum accuracy in diagnosing cardiovascular diseases. Functional diagrams for implementing the proposed method reflecting the features of its application are presented. The quantitative parameters of the core of the computational diagnostic procedure can be determined in advance based on the preliminary statistical data of the ECGs for different heart diseases. That is why the developed method is quite simple in terms of computation (computing complexity, accuracy, computing time, etc.) and can be implemented in medical computer decision systems for monitoring cardiovascular diseases and for their diagnosis in real time. The results of the numerical experiment confirm the high accuracy of the developed method for classifying cardiovascular diseases.

## Introduction

Medical statistics show^1–3^ that currently the main cause of death in the world (more than 30%) is diseases of the cardiovascular system (including among people of working age). Therefore, timely high-precision diagnostics of heart diseases, prevention and treatment at an early stage of the development of the disease acquire exceptional relevance. To improve the accuracy of diagnosing the state of the heart in recent decades, computer systems for automatic analysis^4–6^ of electrocardiographic data obtained during the processing of an electrocardiographic signal have been widely used. Automatic analysis of electrocardiograms is a rather complex theoretical problem. First of all, this is due to the physiological origin of the signal^7–9^, which is the reason for its indeterminacy, diversity, variability, unpredictability, non-stationarity and susceptibility to numerous types of interference.

At present, the analysis process, as a rule, is a study of isopotential and other maps generated from the data obtained using the software supplied with the signal recording apparatus.

In medical practice, the conclusions of cardiologists about patients’ diagnoses have, as usual, qualitative or verbal character and are not always confirmed by enough number of quantitative data. In special or difficult situations with disease recognition, diagnosis errors by young or insufficiently experienced medical doctors are possible, and the real diagnostic process may be significantly extended until a final correct decision about the truth diagnosis.

Decision support systems can seriously help medical doctors in the decision-making processes about the diagnosis of different heart diseases, especially in complicated cases. The most perspective approach is based on the recognizing and diagnosing heart disease using automatic computer processing of the electrocardiograms (ECG) of patients.

In the general case, the change of the ECG parameters can be presented as a random sequence of the signals under processing. Developing new computational methods for such signal processing is an important research problem in creating efficient medical decision support systems.

In this article, the authors consider the possibility of increasing the diagnostic accuracy of cardiovascular diseases by implementing the computational method, which is based on the generalized nonlinear canonical decomposition of a random sequence of the change of cardiogram parameters. The absence of any restrictions on the class of random sequences under study makes it possible to achieve maximum accuracy in diagnosing cardiovascular diseases. The main advantage is that quantitative parameters of the computational diagnostic procedure can be determined in advance based on the preliminary statistical data of the ECGs for different heart diseases. That is why the developed method is quite simple in terms of computation (computing complexity, accuracy, computing time, etc.) and can be implemented in medical computer decision systems for monitoring cardiovascular diseases and for their diagnosis in real time.

Thus, the development of efficient mathematical models and computation methods for identifying the high-accuracy individual characteristics of an electrocardiogram (with subsequent classification), as well as the creation of an automated computer diagnostic support system, is an urgent and important task in “medicine–computer science” multidisciplinary research.

The rest of the article covers multiple aspects related to the topic discussion. “Background and analysis of the related works” section consists of the analysis of the related works in the field of ECG processing. In “Problem statement” section authors formulate the problem statement. “Solution” section deals with the development of the computation method and corresponding mathematical model based on the generalized nonlinear canonical decomposition of a random sequence of the change of cardiogram parameters. “Results of the numerical experiment” section represents the modeling and simulation results for different existing methods and comparative results with the proposed computational method. The paper ends with a conclusion in “Conclusion” section.

## Background and analysis of the related works

Diagnostics of electrocardiograms consist of three successive stages: (a) preliminary processing, (b) feature extraction (normalization), and (c) classification. Let us analyze all these stages consequently.

*Fist stage—Preprocessing* reduces signal measurement noise by smoothing the electrocardiogram signal, reducing drift suppression and baseline deviation. The most common existing methods used to reduce signal noise are (a) second order low pass and (b) high pass Butterworth filters^10^, (c) Daubechies wavelet^11^ and (d) orthogonal wavelet-filter^12^. Besides, for baseline adjustment, such techniques as median filter, linear phase high pass filter, mean median filter and others are used also.

*Second stage—Feature extraction* is an interactive process that includes a series of automatic data transformation procedures. In cases with a large number of measurements-features that describe the characteristics of the input signal, the correlation and factor analysis of data can be used to reduce the dimension of the problem. According to the extraction and analysis methods, the features can be divided into the following categories:

- Temporary features^13^ (these features are described in the time domain, representing amplitude, slope and heart rate);
- Spectral features^14,15^ (features are defined in the frequency domain, account for spectral concentration, normalized spectral moments);
- Time-frequency/wavelet features^16,17^ (features extracted from the results of the wavelet transform applied to the electrocardiogram signal);
- Signs of the complexity of geometric distortions^18^ (these signs include various calculations related to the complexity of the considered segment of the electrocardiogram).

The stage of feature extraction ends with the optimization of their number, which allows reducing the set of redundant functions, reducing computational costs and improving the overall performance of the system. This step uses the following three main categories of feature selection methods: (a) wrapper methods (recursive feature elimination^14^; direct feature selection^19^; genetic algorithms^20^; (b) filter methods (correlation)^21^; Chi-Square^15^; analysis of variance (ANOVA)^22^; ReliefF^23^; (c) built-in methods^24^.

To date, a large number of approaches have been developed to solve the problem of diagnosing cardiovascular diseases using various mathematical methods.

*Third stage—The classification process* can be carried out in several iterations, depending on the chosen recognition scheme. In some cases, the results obtained at this stage require a revision of the entire processing scheme as a whole. The most common classification methods are: discriminant function^25^; cluster analysis^26^; artificial neural network^27^; Naive Bayes classifier^28^; support vector machine^29^; k-Nearest Neighbors (k-NNs)^30^; Decision Trees (DT)^31^.

Several different approaches for ECG analysis are based on a chaos theory^32^, a combination of statistical, geometric, and nonlinear heart rate variability features^32^, a semantic web ontology and heart failure expert system^33^, signal averaging method, multivariate analysis^34^, RPCA—recursive principal component analysis^35^, SPSA—simultaneous perturbation stochastic approximation method^36^, ABT—Amplitude Based Technique FDBT—First Derivative Based Technique, SDBT—Second Derivative Based Technique^37^, Hilbert transform^38^ and others.

At the same time, each of the above-mentioned methods has its drawbacks and limitations. That is why the need to develop new effective methods of medical diagnostics has not lost its relevance.

Thus, the change in the values of the electrocardiogram has a stochastic character; therefore, for the diagnosis of cardiovascular diseases, it is necessary to use methods for recognizing random functions and random sequences.

The main method for recognition of the realizations of random sequences is the Bayes decision rule, according to which a decision about the belonging of the realization to a certain class, for which the posterior probability is maximum, is made. The method is theoretically accurate, however, as well as many of its modifications (the Neyman-Pearson, Wald criterion, etc.^39^) is applied in conditions when the stochastic properties of classes of random sequences are fully known. If the prior probabilities of the classes of random sequences are not known, then equal values are assigned to them and the decision rule is modified into the maximum likelihood criterion. The criterion is especially important when solving problems of recognition, in which unlikely events cannot be excluded from consideration (diagnostics of emergency technical systems, medical diagnostics, etc.). However, for the maximum likelihood method, as well as for the Bayes rule, the problem of approximating the multivariate distribution density for a random sequence with a large number of sampling points remains unresolved.

The use of the canonical expansion of Pugachev^40^ makes it possible to pass the decision rule from a multivariate distribution density to the product of one-dimensional distribution densities of uncorrelated random coefficients. However, this approach is valid only for Gaussian random sequences.

The aim of our study is to eliminate the abovementioned disadvantage and develop a diagnostic method that takes into account non-linear stochastic features of changes in cardiogram parameters.

## Problem statement

The accumulated volume of statistical data on various cardiovascular diseases makes it possible to determine with high accuracy the characteristics $E\left[ {C^{{\xi_{g} }} \left( {i - r_{g - 1} } \right) \cdots C^{{\xi_{2} }} \left( {i - r_{1} } \right)C^{{\xi_{1} }} \left( i \right)} \right],$ $\sum\nolimits_{j = 1}^{g} {\xi_{j} \le N,}$ $\, r_{j} = \overline{1,i - 1,}$ $i = \overline{1,I}$ ($E\left[ {} \right]$-expected value) of random sequences $C\left( {i/k} \right), \, i = \overline{1,I,} \, k = \overline{1,K}$ describing the changes in the values of information signs of an electrocardiogram ($K$ includes ($K - 1$) different diseases and one normal state of a person, $I$—the number of information signs of an electrocardiogram). For example, $C\left( {i/1} \right), \, i = \overline{1,I}$—Marfan syndrome; $C\left( {i/2} \right), \, i = \overline{1,I}$ pulmonary embolism; $C\left( {i/3} \right), \, i = \overline{1,I}$—heart attack; $C\left( {i/4} \right), \, i = \overline{1,I}$—cardiomyopathy;$C\left( {i/5} \right), \, i = \overline{1,I}$—pericardial disease; $C\left( {i/6} \right), \, i = \overline{1,I}$—rheumatic heart disease; $C\left( {i/7} \right), \, i = \overline{1,I}$—stroke; $C\left( {i/8} \right), \, i = \overline{1,I}$—normal state of a person; in this case $K = 8$.

As a result of electrocardiography, a certain sequence of values $c\left( i \right), \, i = \overline{1,I}$ can be obtained. It is necessary to determine to which class $k^{*} \in \left\{ {1,..,K} \right\}$ this realization $c\left( i \right), \, i = \overline{1,I}$ belongs.

## Solution

**Figure 1:**
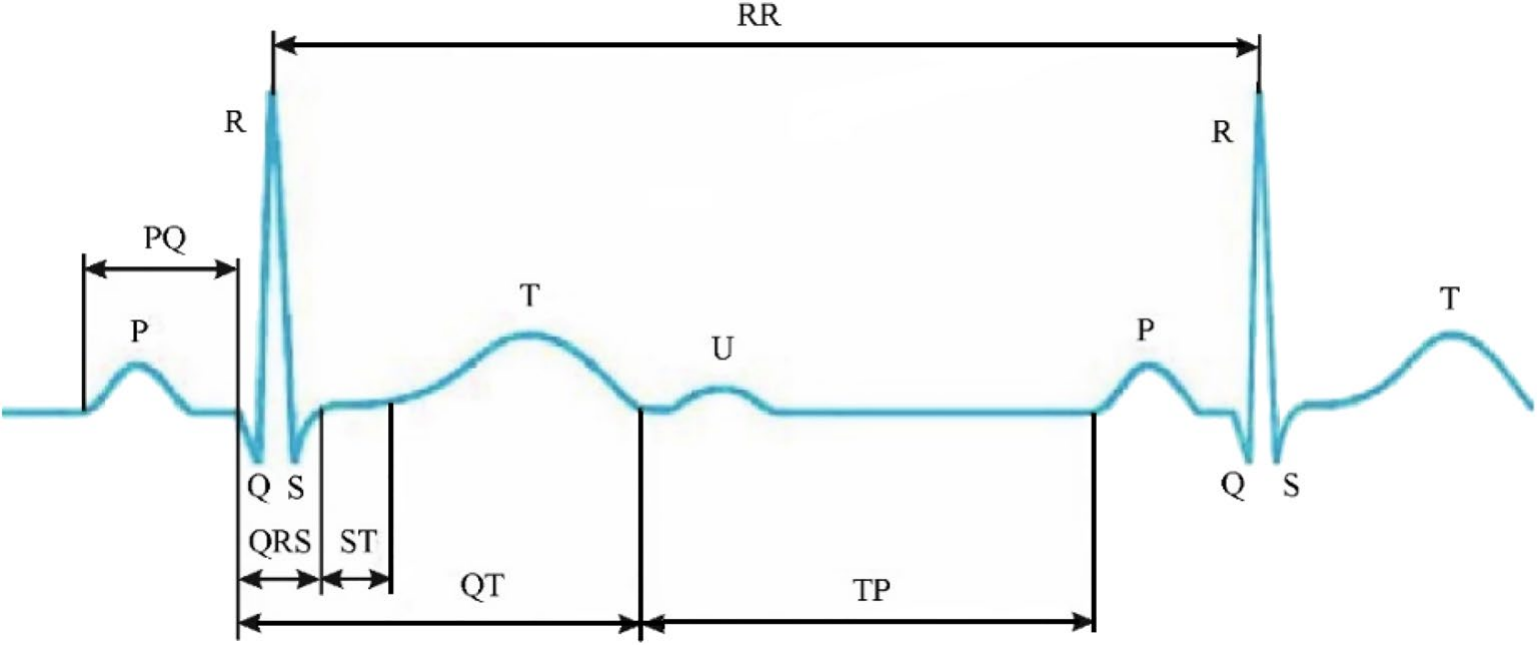
Cardiogram parameters.

Taking into account the specific features of the problem of diagnosing cardiovascular diseases, to analyze the cardiogram, as a rule^41,42^, a random sequence $\left\{ C \right\} = \left\{ {C\left( 1 \right),C\left( 2 \right),...,C\left( I \right)} \right\}, \, I = 14$ with fourteen elements is used. Each element corresponds to one most informative parameter of the electrocardiogram (Fig. 1), in particular: $C\left( 1 \right)$ is the height of the tooth $P$; $C\left( 2 \right)$—the width of the tooth $P$; $C\left( 3 \right)$—the height of the tooth $Q$; $C\left( 4 \right)$—the interval $PQ$; $C\left( 5 \right)$—the height of the first tooth $R$; $C\left( 6 \right)$—the interval $QRS$; $C\left( 7 \right)$—the height of the tooth $S$; $C\left( 8 \right)$—the interval $RR$; $C\left( 9 \right)$—the height of the tooth $T$; $C\left( {10} \right)$—the interval $QT$; $C\left( {11} \right)$—the interval $ST$; $C\left( {12} \right)$—the interval $TP$; $C\left( {13} \right)$—the width of the tooth $U$; $C\left( {14} \right)$—the height of the tooth $U.$

A universal method for recognizing a random sequence is the maximum likelihood criterion^43,44^, according to which the decision on whether the realization $\vec{c} = \left\{ {c\left( 1 \right),...,c\left( {14} \right)} \right\}$ belongs to the class $k^{*} \in \left\{ {1,..,K} \right\}$ is made when the following condition is met^45,46^:

1 $$k^{*} = \arg \mathop {\max }\limits_{k} \left\{ {f_{I} \left( {\vec{c}/k} \right)} \right\}, \, I = 14,$$

where $f_{I} \left( {\vec{c}/k} \right), \, k = \overline{1,K} , \, I = 14$ is the conditional distribution density of the features $\vec{c}$, provided that the realization belongs to this class.

Thus, to solve the recognition problem, it is necessary to obtain the estimate of unknown densities $$f_{I} \left( {\vec{c}/k} \right), \, k = \overline{1,K}$$, which, in turn, taking into account rather large dimension ($$I = 14$$) of the function, is a complex and time-consuming procedure. In the case of using the simplifying assumption ($$E\left[ {C^{\nu } \left( j \right)C^{\mu } \left( i \right)} \right] \ne 0,$$$$E\left[ {C^{{\xi_{g} }} \left( {i - p_{g - 1} } \right)...C^{{\xi_{2} }} \left( {i - p_{1} } \right)C^{{\xi_{1} }} \left( i \right)} \right] = 0$$) about the presence in a random sequence of only stochastic relations between two arbitrary parameters, the problem is greatly simplified by means of transition from the sequence $$C\left( i \right),\,i = \overline{1,14}$$ to the analysis of a set of independent random coefficients $$P_{i}^{(N)} , \, i = \overline{1,14}$$ of the canonical expansion^47,48^:

2 $$C\left( i \right) = E\left[ {C\left( i \right)} \right] + \sum\limits_{\nu = 1}^{i - 1} {\sum\limits_{j = 1}^{N} {P_{\nu }^{(j)} } } T_{\lambda \nu }^{(j)} \left( i \right) + P_{i}^{(1)} ,\quad \, i = \overline{1,14} ;$$

3 $$P_{i}^{(\lambda )} = C^{\lambda } \left( i \right) - \sum\limits_{\nu = 1}^{i - 1} {\sum\limits_{j = 1}^{N} {P_{\nu }^{(j)} } } T_{\lambda \nu }^{(j)} \left( i \right) - \sum\limits_{j = 1}^{\lambda - 1} {P_{i}^{(j)} } T_{\lambda i}^{(j)} \left( i \right),\quad \, i = \overline{1,14} .$$

The coordinate functions $T_{h\nu }^{(\lambda )} \left( i \right)$ are determined from the relation^49,50^:

4 $$\begin{aligned} T_{h\nu }^{(\lambda )} \left( i \right) = & \frac{1}{{D_{\lambda } \left( \nu \right)}}\left( {E\left[ {C^{\lambda } \left( \nu \right)C^{h} \left( i \right)} \right] - E\left[ {C^{\lambda } \left( \nu \right)} \right]E\left[ {C^{h} \left( i \right)} \right] - \sum\limits_{\rho = 1}^{\nu - 1} {\sum\limits_{j = 1}^{N} {D_{j} } } \left( \rho \right)} \right. \\ & \times \,\left. {T_{\lambda \mu }^{(j)} \left( \nu \right)T_{h\rho }^{(j)} \left( i \right) - \sum\limits_{j = 1}^{\lambda - 1} {D_{j} } \left( \nu \right)T_{\lambda \nu }^{(j)} \left( \nu \right)T_{h\nu }^{(j)} \left( i \right)} \right), \, \quad \nu = \overline{1,i} , \, i = \overline{1,14} . \\ \end{aligned}$$

where $D_{\lambda } \left( i \right)$—the variances of the random coefficients $P_{i}^{(\lambda )}$

5 $$\begin{aligned} D_{\lambda } \left( i \right) = & E\left[ {C^{2\lambda } \left( i \right)} \right] - E^{2} \left[ {C^{\lambda } \left( i \right)} \right] - \sum\limits_{\rho = 1}^{i - 1} {\sum\limits_{j = 1}^{N} {D_{j} \left( \rho \right)} } \\ & \times \,\left\{ {T_{\lambda \rho }^{(j)} \left( i \right)} \right\}^{2} - \sum\limits_{j = 1}^{\lambda - 1} {D_{j} \left( i \right)} \left\{ {T_{\lambda i}^{(j)} \left( i \right)} \right\}^{2} ,\quad \, i = \overline{1,14} . \\ \end{aligned}$$

In this case, the substitution of $\vec{c}$ by the vector $\vec{p}$ considering $f_{I} \left( {\vec{p}/k} \right) = \prod\nolimits_{i = 1}^{I} {f_{1} \left( {p_{i}^{(N)} /k} \right)} ,\quad \, k = \overline{1,K} , \, I = 14$ allows us to write the decision rule in the following form

6 $$k^{*} = \arg \mathop {\max }\limits_{j} \prod\limits_{i = 1}^{I} {f_{1} \left( {p_{i}^{(N)} /k} \right)} ,\quad \, k = \overline{1,K} , \, I = 14.$$

The problem of recognition, therefore, is reduced to a successive approximation of twelve one-dimensional distribution densities.

The decision rule (1) is significantly simplified, however, the transition from the vector $\vec{c}$ to the vector $\vec{p}$ is possible provided that the random sequence $C\left( i \right), \, i = \overline{1,14}$ has only stochastic relations $E\left[ {C^{\nu } \left( j \right)C^{\mu } \left( i \right)} \right]$.

To eliminate all existing probabilistic relationships $E\left[ {C^{{\xi_{g} }} \left( {i - r_{g - 1} } \right)...C^{{\xi_{2} }} \left( {i - r_{1} } \right)C^{{\xi_{1} }} \left( i \right)} \right]$, we introduce into consideration the array of random variables:

7 $$C = \left[ {\begin{array}{*{20}c} \begin{gathered} C\left( i \right) \hfill \\ C^{2} \left( i \right) \hfill \\ \end{gathered} \\ \cdots \\ {C^{N - 1} \left( i \right)} \\ {C^{{\xi_{2}^{(2)} }} \left( {i - r_{1}^{(2)} } \right)C^{{\xi_{1}^{(2)} }} \left( i \right)} \\ \cdots \\ {C^{{\xi_{g}^{(g)} }} \left( {i - r_{g - 1}^{(g)} } \right) \cdots C^{{\xi_{2}^{(g)} }} \left( {i - r_{1}^{(g)} } \right)C^{{\xi_{1}^{(g)} }} \left( i \right)} \\ \end{array} } \right].$$

The parameters of the matrix *C* are determined by the expressions

$$i = \overline{1,I} ; \, \xi_{\mu }^{(g)} = \overline{{1,\xi_{\mu }^{\prime (g)} }} , \, \mu = \overline{1,l} ;r_{j}^{(g)} = \overline{{r_{j - 1}^{(g)} + 1,r_{j}^{\prime (g)} }} , \, j = \overline{1,l - 1} ,r_{0}^{(g)} = 0;{\text{ l}} = \overline{1,N - 1} ;$$

$$\begin{gathered} r_{j}^{\prime (g)} = \left\{ {\begin{array}{*{20}l} {0,} \hfill & {{\text{for}}\, \, \left( {j \ne \overline{1,g - 1} } \right) \vee \left( {g = 1} \right);} \hfill \\ {i - g + j,} \hfill & {{\text{for }}\, \, j = \overline{1,g - 1} , \, g > 1;} \hfill \\ \end{array} } \right. \hfill \\ \xi_{\mu }^{\prime (g)} = N - 1 - g + \mu - \sum\limits_{j = 1}^{\mu - 1} {\xi_{j}^{\prime (g)} ,\mu = \overline{1,g} .} \hfill \\ \end{gathered}$$

A priori information $E\left[ {C^{{\xi_{g} }} \left( {i - r_{g - 1} } \right) \cdots C^{{\xi_{2} }} \left( {i - r_{1} } \right)C^{{\xi_{1} }} \left( i \right)} \right]$ about the sequence $C\left( i \right), \, i = \overline{1,14}$ can be obtained by determining the cross-correlation of the elements the array *C*. Consider this array as a vector random sequence $\vec{C}$, each component of which corresponds to a row of the array $C$. Applying the vector linear canonical decomposition to $\vec{C}$ gives the following expression for the first component of (7).

8 $$\begin{aligned} C\left( i \right) = & E\left[ {C\left( i \right)} \right] + \sum\limits_{\nu = 1}^{i - 1} {\sum\limits_{{\xi_{1}^{(1)} = 1}}^{N - 1} {P_{{\xi_{1}^{(1)} }} } \left( \nu \right)\omega \left( {\nu ,\xi_{1}^{(1)} /i;1} \right) + P_{1} \left( i \right)} \\ & + \,\sum\limits_{\nu = 1}^{i - 1} {\sum\limits_{g = 2}^{M(\nu )} {\sum\limits_{{r_{1}^{(g)} = 1}}^{{r_{1}^{\prime (g)} }} { \cdots \sum\limits_{{r_{g - 1}^{(g)} = r_{g - 2}^{(g)} + 1}}^{{r_{g - 1}^{\prime (g)} }} {\sum\limits_{{\xi_{1}^{(g)} = 1}}^{{\xi_{1}^{\prime (g)} }} { \cdots \sum\limits_{{\xi_{g}^{(g)} = 1}}^{{\xi_{g}^{\prime (g)} }} {P_{{r_{1}^{(g)} \cdots r_{g - 1;}^{(g)} \xi_{1}^{(g)} \cdots \xi_{g}^{(g)} }} \left( \nu \right)} } } } } } \\ & \times \,\omega \left( {\nu ;r_{1}^{(g)} \cdots r_{g - 1}^{(g)} ,\xi_{1}^{(g)} \cdots \xi_{g}^{(g)} /i;1} \right),\quad \, i = \overline{1,14} , \\ \end{aligned}$$

where $$M\left( \nu \right) = \left\{ {\begin{array}{*{20}l} {N - 1,} \hfill & {{\text{for }}\,\nu \ge N - 1;} \hfill \\ {\nu ,} \hfill & {{\text{for }}\,\nu < N - 1.} \hfill \\ \end{array} } \right.$$

The coordinate functions $\omega \left( {\nu ;\alpha_{1} /i;b_{1} \cdots b_{m - 1} ;a_{1} \cdots a_{m} } \right)$, $\omega \left( {\nu ;\beta_{1} , \ldots \beta_{n - 1} ;\alpha_{1} , \ldots \alpha_{n} /i;b_{1} , \ldots b_{m - 1} ;a_{1} , \ldots a_{m} } \right)$ of the canonical decomposition (8) are determined by the relations:

9 $$\begin{aligned} \omega \left( {\nu ;\alpha_{1} /i;b_{1} \cdots b_{m - 1} ;a_{1} \cdots a_{m} } \right) = & \,\frac{1}{{D_{{\alpha_{1} }} \left( \nu \right)}}\left\{ {E\left[ {C^{{\alpha_{1} }} \left( \nu \right)C^{{a_{m} }} \left( {i - b_{m - 1} } \right) \cdots C^{{a_{1} }} \left( i \right)} \right]} \right. \\ & - \,E\left[ {C^{{\alpha_{1} }} \left( \nu \right)} \right]M\left[ {C^{{a_{m} }} \left( {i - b_{m - 1} } \right) \cdots C^{{a_{1} }} \left( i \right)} \right] \\ & - \,\sum\limits_{\lambda = 1}^{\nu - 1} {\sum\limits_{{\xi_{1}^{(1)} = 1}}^{N - 1} {D_{{}}^{{_{{\xi_{1}^{\left( 1 \right)} }} }} } \left( \lambda \right)\omega \left( {\lambda ;\xi_{1}^{(1)} /\nu ;\alpha_{1} } \right)\omega \left( {\lambda ;\xi_{1}^{\left( 1 \right)} /i;b_{1} \cdots b_{m - 1} ;a_{1} \cdots a_{m} } \right)} \\ & - \,\sum\limits_{{\xi_{1}^{(1)} = 1}}^{{\alpha_{1} - 1}} {D^{{\xi_{1}^{\left( 1 \right)} }} } \left( \nu \right)\omega \left( {\nu ;\xi_{1}^{\left( 1 \right)} /\nu ;\alpha_{1} } \right)\omega \left( {\nu ;\xi_{1}^{\left( 1 \right)} /\nu ;b_{1} \cdots b_{m - 1} ;a_{1} \cdots a_{m} } \right) \\ & - \,\sum\limits_{\lambda = 1}^{\nu - 1} {\sum\limits_{g = 2}^{M(\lambda )} {\sum\limits_{{r_{1}^{(g)} = 1}}^{{r_{1}^{\prime \left( g \right)} }} { \cdots \sum\limits_{{r_{g - 1}^{(g)} = r_{g - 2}^{(g)} + 1}}^{{r_{g - 1}^{\prime \left( g \right)} }} {\sum\limits_{{\xi_{1}^{(g)} }}^{{\xi_{1}^{\prime \left( g \right)} }} { \cdots \sum\limits_{{\xi_{g}^{(g)} }}^{{\xi_{g}^{\prime \left( g \right)} }} {D_{{}}^{{r_{1}^{\left( g \right)} \cdots r_{g - 1;}^{\left( g \right)} \xi_{1}^{\left( g \right)} \cdots \xi_{g}^{\left( g \right)} }} \left( \lambda \right)} } } } } } \\ & \times \,\omega \left( {\lambda ;r_{1}^{\left( g \right)} \cdots r_{g - 1}^{\left( g \right)} ;\xi_{1}^{\left( g \right)} \cdots \xi_{g}^{\left( g \right)} /\nu ;\alpha_{1} } \right) \\ & \times \,\left. {\omega \left( {\lambda ;r_{1}^{\left( g \right)} \cdots r_{g - 1}^{\left( g \right)} ;\xi_{1}^{\left( g \right)} \cdots \xi_{g}^{\left( g \right)} /\nu ;b_{1} \cdots b_{m - 1} ;a_{1} \cdots a_{m} } \right)} \right\}, \, \quad \nu = \overline{1,14} . \\ \end{aligned}$$

10 $$\begin{aligned} & \omega \left( {\nu ;\beta_{1} , \ldots \beta_{n - 1} ;\alpha_{1} , \ldots \alpha_{n} /i;b_{1} , \ldots b_{m - 1} ;a_{1} , \ldots a_{m}^{{}} } \right) = \,\frac{1}{{D_{{\beta_{1} , \ldots \beta_{n - 1} ;\alpha_{1} , \ldots \alpha_{n}^{{}} }} \left( \nu \right)}} \\ & \quad \quad \left. {\left\{ {E\left[ {C^{{\alpha_{n} }} \left( {\nu - \beta_{n - 1} } \right) \cdots C^{{\alpha_{1} }} \left( \nu \right)} \right.} \right. \times C^{{a_{m} }} \left( {i - b_{m - 1} } \right) \cdots C^{{a_{1} }} \left( i \right)} \right] - M\left[ {C^{{\alpha_{n} }} \left( {\nu - \beta_{n - 1} } \right) \cdots C^{{\alpha_{1} }} \left( \nu \right)} \right] \\ & \quad \times \,E\left[ {C^{{a_{m} }} \left( {i - b_{m - 1} } \right) \cdots C^{{a_{1} }} \left( i \right)} \right] - E\left[ {C^{{\alpha_{n} }} \left( {\nu - \beta_{n - 1} } \right) \cdots C^{{\alpha_{1} }} \left( \nu \right)} \right]E\left[ {C^{{a_{m} }} \left( {i - b_{m - 1} } \right) \cdots C^{{a_{1} }} \left( i \right)} \right] \\ & \quad - \,\sum\limits_{\lambda = 1}^{\nu - 1} {\sum\limits_{{\xi_{1}^{(1)} = 1}}^{N - 1} {D_{{}}^{{\xi_{1}^{(1)} }} } \left( \lambda \right)\omega \left( {\lambda ;\xi_{1}^{(1)} /\nu ;\beta_{1} , \ldots \beta_{n - 1} ;\alpha_{1} ,...\alpha_{n}^{{}} } \right)\omega \left( {\lambda ;\xi_{1}^{(1)} /i;b_{1} \cdots b_{m - 1} ;a_{1} \cdots a_{m} } \right)} \\ & \quad - \,\sum\limits_{\lambda = 1}^{\nu - 1} {\sum\limits_{g = 2}^{M(\lambda )} {\sum\limits_{{r_{1}^{(l)} = 1}}^{{p_{1}^{\prime (g)} }} { \cdots \sum\limits_{{r_{g - 1}^{(g)} = r_{g - 2}^{(g)} + 1}}^{{p_{g - 1}^{\prime (g)} }} {\sum\limits_{{\xi_{1}^{(g)} = 1}}^{{\xi_{1}^{\prime (g)} }} { \cdots \sum\limits_{{\xi_{g}^{(g)} = 1}}^{{\xi_{g}^{\prime (g)} }} {D_{{}}^{{r_{1}^{(g)} \ldots r_{g - 1;}^{(g)} \xi_{1}^{(g)} \ldots \xi_{g}^{(g)} }} \left( \lambda \right)} } } } } } \\ & \quad \times \,\omega \left( {\lambda ;r_{1}^{(g)} \cdots r_{g - 1;}^{(g)} \xi_{1}^{(g)} \cdots \xi_{g}^{(g)} /\nu ;\beta_{1} , \ldots \beta_{n - 1} ;\alpha_{1} , \ldots \alpha_{n}^{{}} } \right) \\ & \quad \times \,\omega \left( {\lambda ;r_{1}^{(g)} \cdots r_{g - 1;}^{(g)} \xi_{1}^{(g)} \cdots \xi_{g}^{(g)} /i;b_{1} , \ldots b_{m - 1} ;a_{1} , \ldots a_{m}^{{}} } \right) \\ & \quad - \,\sum\limits_{l = 2}^{n - 1} {\sum\limits_{{p_{1}^{(g)} = 1}}^{{p_{1}^{\prime (g)} }} { \cdots \sum\limits_{{p_{g - 1}^{(g)} = p_{g - 2}^{(g)} + 1}}^{{p_{g - 1}^{\prime (g)} }} {\sum\limits_{{\xi_{1}^{(g)} = 1}}^{{\xi_{1}^{\prime (g)} }} { \cdots \sum\limits_{{\xi_{g}^{(g)} = 1}}^{{\xi_{g}^{\prime (g)} }} {D_{{}}^{{p_{1}^{(g)} ...p_{g - 1;}^{(g)} \xi_{1}^{(g)} ...\xi_{g}^{(g)} }} \left( \nu \right)} } } } } \\ & \quad \times \,\omega \left( {\nu ;p_{1}^{(g)} \cdots p_{g - 1;}^{(g)} \xi_{1}^{(g)} \cdots \xi_{g}^{(g)} /\nu ;\beta_{1} , \ldots \beta_{n - 1} ;\alpha_{1} , \ldots \alpha_{n}^{{}} } \right) \\ & \quad \times \,\omega \left( {\nu ;b_{1} , \ldots b_{m - 1} ;a_{1} , \ldots a_{m}^{{}} /i;p_{1}^{(g)} \cdots p_{g - 1;}^{(g)} \xi_{1}^{(g)} \cdots \xi_{g}^{(g)} } \right) \\ & \quad - \,\sum\limits_{{r_{1}^{(n)} = 1}}^{{r_{1}^{*(n)} }} { \cdots \sum\limits_{{r_{l - 1}^{(n)} = r_{l - 2}^{(n)} + 1}}^{{r_{n - 1}^{*(n)} }} {\sum\limits_{{\xi_{1}^{(n)} = 1}}^{{\xi_{1}^{*(n)} }} { \cdots \sum\limits_{{\xi_{n}^{(n)} = 1}}^{{\xi_{n}^{*(n)} }} {D_{{}}^{{r_{1}^{(n)} \ldots r_{n - 1;}^{(n)} \xi_{1}^{(n)} \cdots \xi_{n}^{(n)} }} \left( \nu \right)} } } } \\ & \quad \times \,\omega \left( {\nu ;r_{1}^{(n)} \cdots r_{n - 1;}^{(n)} \xi_{1}^{(n)} \cdots \xi_{n}^{(n)} /\nu ;\beta_{1} , \ldots \beta_{n - 1} ;\alpha_{1} , \ldots \alpha_{n}^{{}} } \right) \\ & \quad \times \,\left. {\omega \left( {\nu ;r_{1}^{(n)} \cdots r_{n - 1;}^{(n)} \xi_{1}^{(n)} \cdots \xi_{n}^{(n)} /i;b_{1} ,...b_{m - 1} ;a_{1} ,...a_{m}^{{}} } \right)} \right\},\quad \, \nu = \overline{1,14} ; \\ \end{aligned}$$

where $D^{{\alpha_{1} }} \left( \nu \right)$, $D_{{}}^{{\beta_{1} ,...\beta_{n - 1} ;\alpha_{1} ,...\alpha_{n}^{{}} }} \left( \nu \right)$—variances of random coefficients $P_{{\alpha_{1} }} \left( \nu \right)$, $P_{{\beta_{1} ,...\beta_{n - 1} ;\alpha_{1} ,...\alpha_{n}^{{}} }} \left( \nu \right)$.

The values $b_{1} ,...,b_{m - 1} ;{\text{ a}}_{1} ,...,a_{m}$ change in the intervals $b_{\mu } \in \left[ {b_{\mu - 1}^{(m)} ;b_{\mu }^{^{\prime}(m)} } \right] \,$ and $a_{\mu } \in \left[ {1;a_{\mu }^{\prime (m)} } \right]{, }m{ = }\overline{1 - N} \,$ respectively. The right boundaries of the intervals are determined from the formulas

$$\begin{aligned} & b_{\mu }^{\prime \left( m \right)} = \left\{ {\begin{array}{*{20}l} {0,} \hfill & {\left( {\mu \ne \overline{1,m - 1} } \right) \vee \, \left( {m = 1} \right);} \hfill \\ {i - m + \mu ,} \hfill & {\mu = \overline{1,m - 1} , \, m > 1;} \hfill \\ \end{array} } \right. \\ & a_{\mu }^{\prime \left( m \right)} = N - 1 - m + \mu - \sum\limits_{j = 1}^{\mu - 1} {a_{j}^{(m)} ,\mu = \overline{1,m} .} \\ \end{aligned}$$

**Figure 2:**
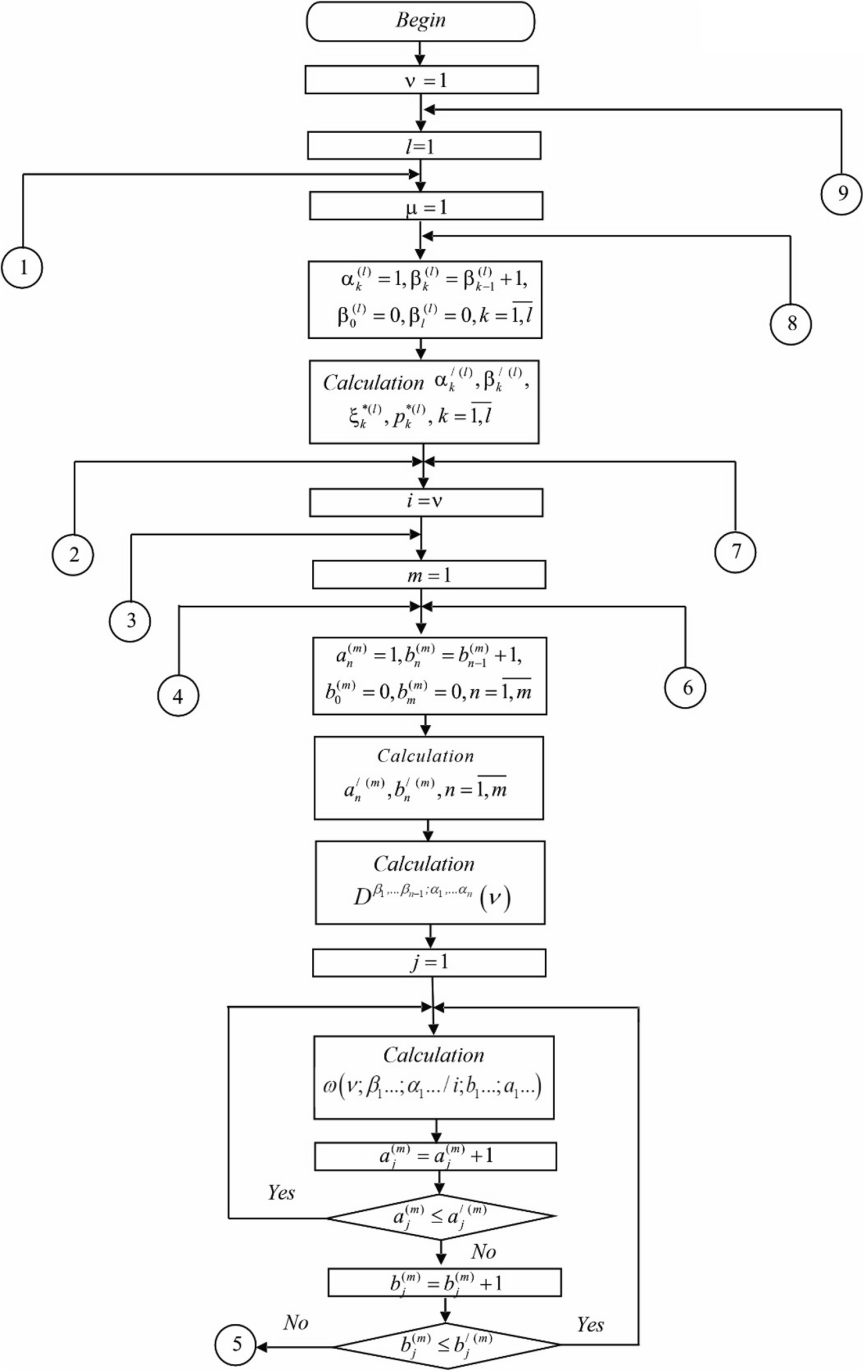

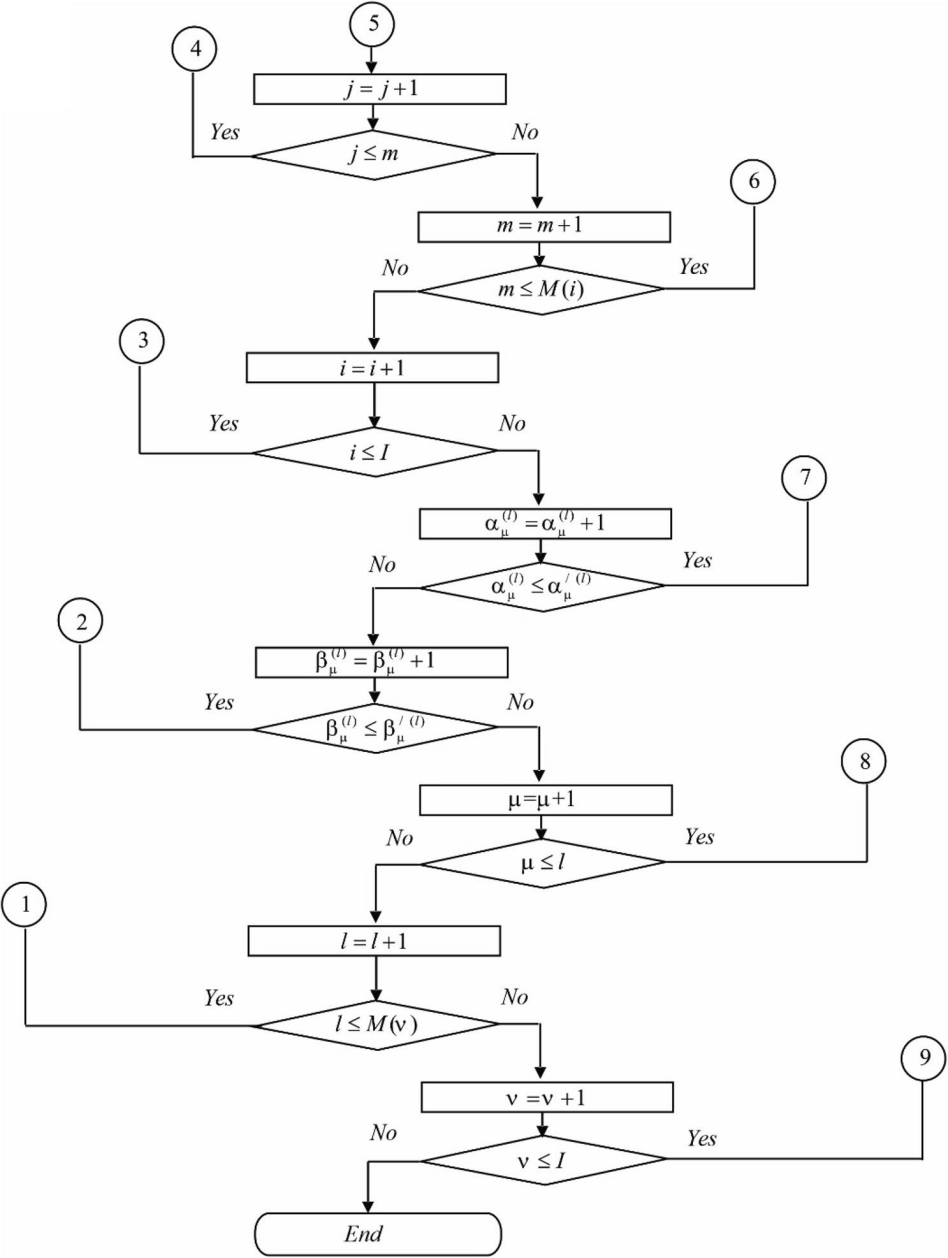
Block diagram of the algorithm for calculating the parameters of the canonical expansion (8).

The block diagram of the algorithm for calculating the parameters of the canonical expansion (8) is shown in Fig. 2.

Expression (8) is a non-linear canonical decomposition of the investigated sequence with full consideration of the stochastic properties $E\left[ {C^{{\xi_{g} }} \left( {i - r_{g - 1} } \right)...C^{{\xi_{2} }} \left( {i - r_{1} } \right)C^{{\xi_{1} }} \left( i \right)} \right].$ Therefore, the random coefficients $P_{N - 1} \left( 1 \right)$, $P_{1;1,N - 2} \left( 2 \right)$, $P_{1,2;1,1,N - 3} \left( 3 \right)$, …, $P_{14 - N + 1,14 - N + 2,...,11;1,1...1} \left( {14} \right)$, calculated at the last iteration, are independent random variables and the decision rule takes the form:

11 $$k^{*} = \arg \mathop {\max }\limits_{k} \left\{ {f_{1} \left( {p_{N - 1} \left( 1 \right)} \right)f_{1} \left( {p_{1;1,N - 2} \left( 2 \right)} \right)...f_{1} \left( {p_{14 - N + 1,14 - N + 2,...,13;1,1...1} \left( {14} \right)} \right)/k, \, k = \overline{1,K} } \right\}$$

The absence of assumptions about the form of the distribution density of random variables $P_{N - 1} \left( 1 \right)$, $P_{1;1,N - 2} \left( 2 \right)$, $P_{1,2;1,1,N - 3} \left( 3 \right)$, …, $P_{14 - N + 1,14 - N + 2,...,13;1,1...1} \left( {14} \right)$ leads to the need to use nonparametric methods to describe them. The simplest and most efficient approach under these conditions is the use of nonparametric Parzen-type estimates^51^.

*The computational method for diagnosing cardiovascular diseases consists in the realization of the following stages*:

1. Estimation of moment functions $$E\left[ {C^{{\xi_{g} }} \left( {i - r_{g - 1} } \right) \ldots C^{{\xi_{2} }} \left( {i - r_{1} } \right)C^{{\xi_{1} }} \left( i \right)} \right],$$ $$\sum\limits_{j = 1}^{g} {\xi_{j} \le N,}$$$$r_{j} = \overline{1,i - 1,}$$$$i = \overline{1,14}$$ based on statistical information $$c_{l} \left( i \right), \, i = \overline{1,14} ,{\text{ l}} = \overline{1,L}$$;
2. Formation of canonical decompositions (8) for various classes (diseases) of random sequences;
3. Synthesis of one-dimensional distribution densities of independent random coefficients $P_{N - 1} \left( 1 \right)$, $P_{1;1,N - 2} \left( 2 \right)$, $P_{1,2;1,1,N - 3} \left( 3 \right)$, …, $P_{14 - N + 1,14 - N + 2, \ldots ,13;1,1 \ldots 1} \left( {14} \right)$;
4. Calculation of the values $p_{N - 1} \left( 1 \right)$, $p_{1;1,N - 2} \left( 2 \right)$, $p_{1,2;1,1,N - 3} \left( 3 \right)$, …, $p_{14 - N + 1,14 - N + 2, \ldots ,13;1,1 \ldots 1} \left( {14} \right)$ for some cardiogram $\vec{c} = \left\{ {c\left( 1 \right), \ldots ,c\left( {14} \right)} \right\}$;
5. Determination of the belonging of the cardiogram $\vec{c} = \left\{ {c\left( 1 \right), \ldots ,c\left( {14} \right)} \right\}$ to a certain class based on the decision rule (11).

**Figure 3:**
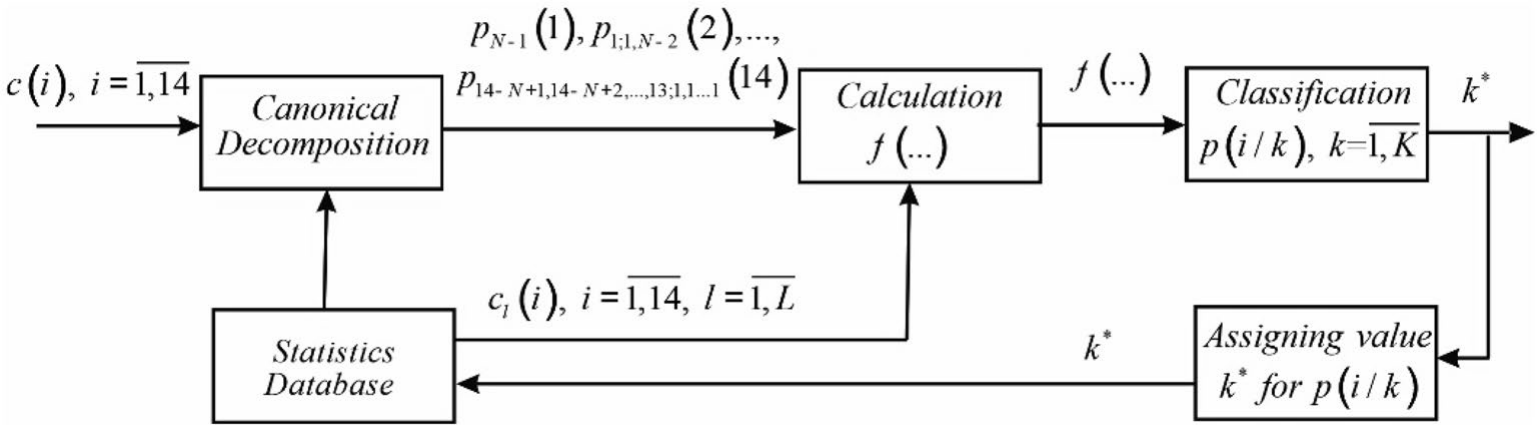
Diagram of the functioning of the system of diagnostics of cardiovascular diseases.

The diagram of the functioning of the system of diagnostics of cardiovascular diseases based on the developed method is shown in Fig. 3.

## Results of the numerical experiment

The proposed method was tested on the basis of statistical data of nine cardiovascular diseases: (a) mild neurocirculatory dystonia, $\left\{ {C\left( i \right)/1} \right\}, \, i = \overline{1,14}$; (b) neurocirculatory dystonia of moderate degree, $\left\{ {C\left( i \right)/2} \right\}, \, i = \overline{1,14}$; (c) severe neurocirculatory dystonia, $\left\{ {C\left( i \right)/3} \right\}, \, i = \overline{1,14}$; (d) stenocardia of the first functional class, $\left\{ {C\left( i \right)/4} \right\}, \, i = \overline{1,14}$;

(e) hypertrophy of myocardium, $\left\{ {C\left( i \right)/5} \right\}, \, i = \overline{1,14}$; (f) severe arrhythmia, $\left\{ {C\left( i \right)/6} \right\}, \, i = \overline{1,14}$; (g) aortic stenosis, $\left\{ {C\left( i \right)/7} \right\}, \, i = \overline{1,14}$; (h) stenocardia of the second functional class, $\left\{ {C\left( i \right)/8} \right\}, \, i = \overline{1,14}$; (i) stenocardia of the third functional class, $\left\{ {C\left( i \right)/9} \right\}, \, i = \overline{1,14}$.

For the numerical experiment two hundred different cardiograms for each disease $\left\{ {C\left( i \right)/k} \right\}, \, i = \overline{1,14} , \, k = \overline{1,K} , \, K = 9$ were used from the Physikalisch-Technische Bundesanstalt (PTB) dataset, which is a publicly available database. This database is compiled by the National Metrology Institute of Germany. It contains combinations of digitized ECGs of both normal and abnormal subjects’ recordings, which are provided for research^52^.

Testing the statistical hypothesis about the independence of random coefficients $$P_{N - 1} \left( 1 \right)$$, $$P_{1;1,N - 2} \left( 2 \right)$$, $$P_{1,2;1,1,N - 3} \left( 3 \right)$$, …, $$P_{14 - N + 1,14 - N + 2, \ldots ,13;1,1.. \ldots 1} \left( {14} \right)$$ based on the criterion $$\chi^{2}$$^53^ and the Bloom criterion^54^, showed the truth of the hypothesis about the independence of coefficients at $$N = 4$$ for all six sequences with a probability not less than $$P_{D} = 0,98$$ (for a linear decomposition there is a statistically significant relationship between random coefficients; for $$N = 3$$, there are not enough grounds for accepting the hypothesis about independence of random coefficients for $$i = 3, \, i = 6, \, i = 7, \, i = 8$$). Thus, the canonical expansion (8) for $$N = 4$$ (random coefficients are a array of independent random variables) with the corresponding sets of coordinate functions are adequate models^40^ of the studied random sequences $$\left\{ {C\left( i \right)/k} \right\}, \, i = \overline{1,14} , \, k = \overline{1,9}$$. The following methods were used for recognition: (a) linear criterion (rule (6) for $$E\left[ {C\left( j \right)C\left( i \right)} \right] \ne 0,$$$$E\left[ {C^{\nu } \left( j \right)C^{\mu } \left( i \right)} \right] = 0, \, \nu + \mu > 2$$); (b) polynomial criterion (6) ^55^; (c) fuzzy logic method ^56–58^; (d) neural network ^59,60^ based on the Daubechies wavelet function of the fourth order and the Levenberg–Marquardt algorithm for learning; (e) generalized non-linear criterion (11). Let us consider mentioned criteria in details:

- *Linear criterion*
  12 $$k^{*} = \arg \mathop {\max }\limits_{k} \prod\limits_{i = 1}^{14} {f_{1} \left( {p_{i}^{{}} /k} \right)} ,\quad \, k = \overline{1,6} ,$$
  where $p_{i}^{{}} , \, i = \overline{1,I} , \, I = 14$ are random values of uncorrelated random coefficients $P_{i}^{{}} , \, i = \overline{1,14} :$
  $$P_{i} = C\left( i \right) - E\left[ {C\left( i \right)} \right] - \sum\limits_{\nu = 1}^{i - 1} {P_{\nu } T_{\nu } \left( i \right)} ,\quad \, i = \overline{1,14} .$$
  Coordinate functions
  $$\begin{aligned} T_{\nu } \left( i \right) & = \frac{1}{{D_{{}} \left( \nu \right)}}\left( {E\left[ {C\left( \nu \right)C\left( i \right)} \right] - E\left[ {C\left( \nu \right)} \right]E\left[ {C\left( i \right)} \right] - \sum\limits_{\rho = 1}^{\nu - 1} {D\left( \rho \right)} } \right. \\ & \times \left. {T_{\rho } \left( \nu \right)T_{\rho } \left( i \right)} \right),\quad \nu = \overline{1,i} ,\quad i = \overline{1,14} . \\ \end{aligned}$$
  Variances of random coefficients
  $$D_{\lambda } \left( i \right) = E\left[ {C^{2\lambda } \left( i \right)} \right]{ - }E^{2} \left[ {C^{\lambda } \left( i \right)} \right]{ - }\sum\limits_{\rho = 1}^{\lambda - 1} {D\left( \rho \right)} \left\{ {T_{\rho }^{{}} \left( i \right)} \right\}^{2} ,\quad \, i = \overline{1,14} .$$
- *Polynomial criterion*
  13 $$k^{*} = \arg \mathop {\max }\limits_{j} \prod\limits_{i = 1}^{14} {f_{1} \left( {p_{i}^{(3)} /k} \right)} , \, \quad k = \overline{1,6} .$$
  Random coefficients
  $$P_{i}^{(\lambda )} = C^{\lambda } \left( i \right) - \sum\limits_{\nu = 1}^{i - 1} {\sum\limits_{j = 1}^{3} {P_{\nu }^{(j)} } } T_{\lambda \nu }^{(j)} \left( i \right) - \sum\limits_{j = 1}^{\lambda - 1} {P_{i}^{(j)} } T_{\lambda i}^{(j)} \left( i \right),\quad \, i = \overline{1,14} .$$
  Coordinate functions
  $$\begin{aligned} T_{h\nu }^{(\lambda )} \left( i \right) = & \,\frac{1}{{D_{\lambda } \left( \nu \right)}}\left( {E\left[ {C^{\lambda } \left( \nu \right)C^{h} \left( i \right)} \right] - E\left[ {C^{\lambda } \left( \nu \right)} \right]E\left[ {C^{h} \left( i \right)} \right] - \sum\limits_{\rho = 1}^{\nu - 1} {\sum\limits_{j = 1}^{3} {D_{j} } } \left( \rho \right)} \right. \\ & \times \,\left. {T_{\lambda \mu }^{(j)} \left( \nu \right)T_{h\rho }^{(j)} \left( i \right) - \sum\limits_{j = 1}^{\lambda - 1} {D_{j} } \left( \nu \right)T_{\lambda \nu }^{(j)} \left( \nu \right)T_{h\nu }^{(j)} \left( i \right)} \right),\quad \nu = \overline{1,i} ,\quad \, i = \overline{1,14} . \\ \end{aligned}$$
  Variances of random coefficients
  $$\begin{aligned} D_{\lambda } \left( i \right) = & \,E\left[ {C^{2\lambda } \left( i \right)} \right]{ - }E^{2} \left[ {C^{\lambda } \left( i \right)} \right]{ - }\sum\limits_{\rho = 1}^{{i{ - }1}} {\sum\limits_{j = 1}^{3} {D_{j} \left( \rho \right)} } \\ & \times \,\left\{ {T_{\lambda \rho }^{(j)} \left( i \right)} \right\}^{2} { - }\sum\limits_{j = 1}^{{\lambda { - }1}} {D_{j} \left( i \right)} \left\{ {T_{\lambda i}^{(j)} \left( i \right)} \right\}^{2} ,\quad \, i = \overline{1,14} . \\ \end{aligned}$$
- *Fuzzy logic method for medical diagnostics*
  This method is based on the implementation of fuzzy system with hierarchical structure of Rule Base ^56^.

Input parameters: $p_{1}$—increase of double product per one kilogram of the body weight of the sick; $p_{2}$—increase of double product per one kilogram of physical exertion; $p_{3}$—coefficient of phosphorilation; $p_{4}$—age of the sick; $p_{5}$—double product of pulse on arterial tension; $p_{6}$—adenosinetriphosphoric acid; $p_{7}$—adenosine diphosphoric acid; $p_{8}$—adenylic acid; $p_{9}$—coefficient of the ratio of lactic and pyruvic acid content;$p_{10}$—maximal consumption of oxygen per one kilogram of the body weight of the sick; $p_{11}$—increase of double product in the response for submaximal physical exertion; $p_{12}$—tolerance to physical activity.

Expressions for the determination of the diagnosis are of the form:

14 $$\begin{aligned} & c = f_{c} \left( {p_{1} ,p^{(1)} ,p^{(2)} } \right), \\ & p^{(1)} = f_{{p^{(1)} }} \left( {p_{2} ,p_{3} ,p_{4} ,p_{5} ,p_{10} ,p_{11} } \right), \\ & p^{(2)} = f_{{p^{(2)} }} \left( {p_{6} ,p_{7} ,p_{8} ,p_{9} ,p_{12} } \right) \\ \end{aligned}$$

where values *c*: $c_{1}$—mild neurocirculatory dystonia, $\left\{ {C\left( i \right)/1} \right\}, \, i = \overline{1,14}$; $c_{2}$—neurocirculatory dystonia of moderate degree, $\left\{ {C\left( i \right)/2} \right\}, \, i = \overline{1,14}$; $c_{3}$- severe neurocirculatory dystonia, $\left\{ {C\left( i \right)/3} \right\}, \, i = \overline{1,14}$; $c_{4}$—stenocardia of the first functional class, $\left\{ {C\left( i \right)/4} \right\}, \, i = \overline{1,14}$; $c_{5}$- hypertrophy of myocardium, $\left\{ {C\left( i \right)/5} \right\}, \, i = \overline{1,14}$; $c_{6}$—severe arrhythmia, $\left\{ {C\left( i \right)/6} \right\}, \, i = \overline{1,14}$; $c_{7}$- aortic stenosis, $\left\{ {C\left( i \right)/7} \right\}, \, i = \overline{1,14}$; $c_{8}$—stenocardia of the second functional class, $\left\{ {C\left( i \right)/8} \right\}, \, i = \overline{1,14}$; $c_{9}$—stenocardia of the third functional class, $\left\{ {C\left( i \right)/9} \right\}, \, i = \overline{1,14}$. Tables 1, 2 and 3 are the information base for the formation of a system of fuzzy logic equations that connect the membership functions of the diagnosis and input variables.

**Table 1:** Knowledge base according to equation $c = f_{c} \left( {p_{1} ,p^{(1)} ,p^{(2)} } \right)$.

$p_{1}$	$p^{(1)}$	$p^{(2)}$	$c$
L	L	L	$c_{1}$
L	BA	BA	$c_{1}$
BA	BA	L	$c_{1}$
BA	BA	BA	$c_{2}$
A	BA	BA	$c_{2}$
BA	BA	A	$c_{2}$
A	BA	A	$c_{3}$
AA	AA	BA	$c_{3}$
AA	A	A	$c_{3}$
AA	A	AA	$c_{4}$
A	AB	AA	$c_{4}$
BA	AA	AA	$c_{4}$
BA	A	AA	$c_{5}$
BA	H	AA	$c_{5}$
BA	A	A	$c_{5}$
BA	H	A	$c_{6}$
BA	A	H	$c_{6}$
BA	H	H	$c_{6}$
A	AA	AA	$c_{7}$
AA	AA	AA	$c_{7}$
H	A	A	$c_{7}$
A	H	A	$c_{8}$
AA	AA	H	$c_{8}$
H	AA	AA	$c_{8}$
H	H	H	$c_{9}$
AA	H	AA	$c_{9}$
A	H	AA	$c_{9}$

**Table 2:** Knowledge base according to equation $p^{(1)} = f_{{p^{(1)} }} \left( {p_{2} ,p_{3} ,p_{4} ,p_{5} ,p_{10} ,p_{11} } \right)$.

$p_{2}$	$p_{3}$	$p_{4}$	$p_{5}$	$p_{10}$	$p_{11}$	$p^{(1)}$
H	H	H	L	H	H	L
H	AA	H	BA	H	H	L
AA	H	AA	L	H	H	L
AA	AA	H	BA	H	AA	BA
H	H	AA	A	H	H	BA
AA	AA	H	BA	AA	AA	BA
A	A	A	A	A	A	A
AA	AA	A	BA	AA	A	A
A	AA	AA	A	AA	AA	A
BA	A	BA	AA	BA	BA	AA
BA	BA	A	A	L	BA	AA
A	BA	BA	AA	BA	A	AA
L	L	L	AA	L	L	H
BA	L	BA	H	L	BA	H
L	BA	BA	AA	L	L	H

**Table 3:** Knowledge base according to equation $p^{(2)} = f_{{p^{(1)} }} \left( {p_{6} ,p_{7} ,p_{8} ,p_{9} ,p_{12} } \right)$.

$p_{6}$	$p_{7}$	$p_{8}$	$p_{9}$	$p_{12}$	$p^{(2)}$
H	H	H	H	H	L
AA	H	AA	AA	AA	L
H	AA	H	A	AA	L
AA	AA	A	A	AA	BA
A	AA	A	AA	H	BA
A	H	AA	AA	AA	BA
A	A	A	AA	AA	A
AA	AA	A	A	A	A
AA	A	AA	AA	A	A
BA	A	BA	A	A	AA
AA	BA	A	BA	BA	AA
L	A	A	BA	A	AA
L	L	L	L	BA	H
BA	L	BA	L	L	H
L	BA	BA	L	BA	H

Tables 1, 2, 3 use the abbreviation for fuzzy terms: L—low, BA—below the average, A—average, AA—above average, H—high.

For example, if $p_{1}$ = A, $p^{(1)}$ = H, $p^{(2)}$ = AA, according to the last line of Table 1, the diagnosed disease is stenocardia of the third functional class.

The value H for parameter $p^{(1)}$ is accepted in three cases (rule base Table 2):

- $$p_{2}$$ = L,$$p_{3}$$ = L,$$p_{4}$$ = L,$$p_{5}$$ = AA,$$p_{10}$$ = L,$$p_{11}$$ = L;
- $p_{2}$ = BA,$p_{3}$ = L,$p_{4}$ = BA,$p_{5}$ = H,$p_{10}$ = L,$p_{11}$ = BA;
- $p_{2}$ = L,$p_{3}$ = BA,$p_{4}$ = BA,$p_{5}$ = AA,$p_{10}$ = L,$p_{11}$ = L.

Parameter $p^{(2)}$ is equal to AA if one of the conditions is met (rule base Table 3):

- $$p_{6}$$ = BA, $$p_{7}$$ = A, $$p_{8}$$ = BA, $$p_{9}$$ = A, $$p_{12}$$ = A;
- $p_{6}$ = AA, $p_{7}$ = BA, $p_{8}$ = A, $p_{9}$ = BA, $p_{12}$ = BA;
- $$p_{6}$$ = L, $$p_{7}$$ = A, $$p_{8}$$ = A, $$p_{9}$$ = BA, $$p_{12}$$ = A.

(iv) *Neural network*
  Daubechies wavelet function of the fourth order and the Levenberg–Marquardt algorithm for learning were used ^59^.
  Expressions for the determination of approximation coefficients and detailing of discrete wavelet transform are presented in the form:
  $$\begin{aligned} & W_{\gamma } \left( {j_{0} ,k} \right) = \frac{1}{M}\sum\limits_{x} {F\left( x \right)} \gamma_{{j_{0} ,k}} \left( x \right), \\ & W_{\phi } \left( {j,k} \right) = \frac{1}{M}\sum\limits_{x} {F\left( x \right)} \phi_{j,k} \left( x \right), \\ \end{aligned}$$
  where $\gamma_{j,k} \left( x \right), \, \phi_{j,k} \left( x \right)$ is a family of basic functions.

Output signal of each of separate neuron of output layer was forming as

$$y\left( k \right) = \frac{1}{M}F\left( {\sum\limits_{i = 0}^{K} {w_{ki} } F\left( {\sum\limits_{j = 0}^{N} {w_{ij} x} } \right)} \right)$$

Continuous sigmoid bipolar function $F\left( x \right) = th\left( x \right)$ was used as activation function of each separate neuron.

Tables 4, 5 shows the comparative diagnostic results.

**Table 4:** The results of the diagnostics of cardiovascular diseases $\left\{ {C\left( i \right)/k} \right\}, \, k = \overline{1,5}$ (% of correct diagnoses).

Heading level	$\left\{ {C\left( i \right)/1} \right\}$(%)	$\left\{ {C\left( i \right)/2} \right\}$(%)	$\left\{ {C\left( i \right)/3} \right\}$(%)	$\left\{ {C\left( i \right)/4} \right\}$(%)	$\left\{ {C\left( i \right)/5} \right\}$(%)
Linear criterion (12)	79	83	89	77	82
Polynomial criterion (13)	92	93	96	91	95
Fuzzy logic method (14)	87	89	93	87	92
Neural network	86	87	91	89	94
Non-linear criterion (11)	99	99	100	99	100

**Table 5:** The results of the diagnostics of cardiovascular diseases $\left\{ {C\left( i \right)/k} \right\}, \, k = \overline{6,9}$ (% of correct diagnoses).

Heading level	$\left\{ {C\left( i \right)/6} \right\}$(%)	$\left\{ {C\left( i \right)/7} \right\}$(%)	$\left\{ {C\left( i \right)/8} \right\}$(%)	$\left\{ {C\left( i \right)/9} \right\}$(%)
Linear criterion (12)	85	86	81	84
Polynomial criterion (13)	95	96	93	96
Fuzzy logic method (14)	93	94	88	93
Neural network	93	92	89	94
Non-linear criterion (11)	100	100	100	100

The data in Tables 4, 5 indicate the low efficiency of the linear criterion (12) (the minimum amount of a priori information is used:$E\left[ {C\left( j \right)C\left( i \right)} \right]$). The use of additional stochastic relations ($E\left[ {C^{\nu } \left( j \right)C^{\mu } \left( i \right)} \right]$) in the criterion (13) makes it possible to achieve an increase in the accuracy of solving the problem of diagnosing cardiovascular diseases compared to (12). The maximum accuracy of diagnostics is achieved by applying the proposed decision rule (11) by maximizing the use of the stochastic properties ($E\left[ {C^{{\xi_{l} }} \left( {i - r_{l - 1} } \right) \cdots C^{{\xi_{2} }} \left( {i - r_{1} } \right)C^{{\xi_{1} }} \left( i \right)} \right]$) of the studied random sequences. The existing set of wavelet functions and the lack of a rigorous mathematical apparatus for analyzing fuzzy equations significantly limit the quality of decision-making about a cardiovascular disease based on the fuzzy logic method and neural network.

## Conclusion

A computational method for computer systems for automated diagnosis of cardiovascular diseases based on a generalized nonlinear canonical decomposition of a random sequence of change of cardiograms has been obtained. The use of the canonical model made it possible to form the decision rule for the maximum distribution density in the form of a product of one-dimensional distribution densities of random coefficients. The canonical decomposition does not impose any significant restrictions (linearity, stationarity, Markov property, monotony, ergodicity, etc.) on the class of random sequences under study, which makes it possible to maximally take into account the stochastic characteristics of sequences related to various cardiovascular diseases.

Taking into account the recurrent regularity of calculations, the diagnostic method is quite simple in terms of computation and allows using an arbitrary number of input parameters. A significant advantage of the method is the ability to use characteristics not directly related to the cardiogram (age of the patient, blood pressure, etc.).

During the operation of the diagnostic system based on the proposed computational method, new diseases unknown to medicine can be identified in the case of a significant difference in the values of the likelihood function for the investigated cardiogram and the classified cardiograms of known diseases.

The results of the numerical experiment indicate a high reliability of the diagnostics of cardiovascular diseases based on the proposed method.

## Data Availability

The datasets generated and analysed during the current study are available in the Physikalisch-Technische Bundesanstalt (PTB) repository, https://physionet.org/physiobank/database/.
